# An Automated Tongue Tracker for Quantifying Bulbar Function in ALS

**DOI:** 10.3389/fneur.2022.838191

**Published:** 2022-02-25

**Authors:** Alicia Northall, Budhaditya Mukhopadhyay, Miriam Weber, Susanne Petri, Johannes Prudlo, Stefan Vielhaber, Stefanie Schreiber, Esther Kuehn

**Affiliations:** ^1^Institute for Cognitive Neurology and Dementia Research, Otto-von-Guericke University Magdeburg, Magdeburg, Germany; ^2^Department of Neurology, Otto-von-Guericke University Magdeburg, Magdeburg, Germany; ^3^Department of Neurology, Hannover Medical School, Hanover, Germany; ^4^Department of Neurology, Rostock University Medical Centre, Rostock, Germany; ^5^German Center for Neurodegenerative Diseases (DZNE), Rostock, Germany; ^6^German Center for Neurodegenerative Diseases (DZNE), Magdeburg, Germany; ^7^Center for Behavioral Brain Sciences, Magdeburg, Germany

**Keywords:** tongue, bulbar, amyotrophic lateral sclerosis, neural network, quantification

## Abstract

**Introduction:**

Bulbar symptoms, including difficulty swallowing and speaking, are common in amyotrophic lateral sclerosis (ALS) and other neurological disorders, such as stroke. The presence of bulbar symptoms provides important information regarding clinical outcomes, such as survival time after diagnosis. Nevertheless, there are currently no easily accessible, quantitative methods to measure bulbar function in patients.

**Methods:**

We developed an open-source tool called Tongue Tracker (TT) to quantify bulbar function by training a neural network to track kinematic tongue features of short video clips of lateral tongue movements. We tested 16 healthy controls and ten patients with ALS, of whom two patients were clinically diagnosed with bulbar-onset type and eight patients were clinically diagnosed with limb-onset type. Of the limb-onset patients, five patients also showed symptoms of bulbar impairment.

**Results:**

We validated TT by comparing the results with manual delineation of tongue movements in the clips. We demonstrate an early-stage bulbar-onset patient who showed fewer and slower tongue sweeps compared to healthy controls and limb-onset patients and we show that five bulbar-impaired limb-onset patients have a different tongue kinematic profile compared to healthy controls.

**Discussion:**

TT may serve to detect quantitative markers of bulbar dysfunction in ALS and other motor disorders, such as stroke, by identifying signatures of spasticity or muscle weakness that affects tongue movement speed and/or tongue movement topography.

## Introduction

Bulbar symptoms, characterized by difficulty swallowing and speaking, are common in amyotrophic lateral sclerosis (ALS) and other neurological disorders, such as stroke (1, 2). Critically, the presence of bulbar symptoms is associated with poorer clinical outcomes in ALS, including shorter survival time (3, 4). In ALS, these symptoms are caused by the degeneration of upper and lower motor neurons that support the bulbar muscles in the tongue and throat. While only one-third of patients with ALS first present with bulbar symptoms (so-called “bulbar-onset ALS”), the majority of patients with lower or upper limb-onset ALS will experience bulbar impairment with disease progression because the disorder spreads through the topographically-organized motor system (5–7). Difficulty swallowing, clinically termed dysphagia, causes malnutrition and even respiratory infections in patient populations. The presence of bulbar symptoms is, therefore, a critical marker of both the disease onset and disease progression in ALS and in other neurological disorders including stroke (1, 8). Despite the clinical relevance of bulbar impairment, there is currently no standardized and accessible measure to quantify bulbar function in a clinical setting (2). This study introduces a tool named “Tongue Tracker” (TT), a novel and automated tool for quantifying bulbar function based on short video clips of tongue movements, which can be acquired in the clinic by using a laptop or mobile phone. We first demonstrate the usage of TT in a sample of healthy controls and patients with ALS with and without clinical bulbar symptom manifestation.

In clinical practice, the bulbar sub-score of the ALS Functional Rating Scale-Revised (ALSFRS-R) (9) is the most commonly used measure of bulbar symptoms. While this score correlates with disease progression in ALS (10), it relies on limited, qualitative self-reports of speech intelligibility, salivation levels and swallowing. Even though the Center for Neurologic Study-Bulbar Function Scale (CNS-BFS) (11) is more comprehensive and specific, it is also dependent on subjective self-reports and, therefore, not objective. Alternatively, speech-based measures can identify bulbar symptoms undetected by the ALSFRS-R and clinicians' speech ratings (12). While promising, measures of speech intelligibility are unlikely to support early detection of bulbar impairment since such changes present later in the disease course (13). Dysphagia presents earlier in bulbar impairment, yet advanced measures of swallowing function have not been validated in ALS and require invasive and expensive methods unsuitable in a routine clinical investigation (8).

Interestingly, kinematic features of the articulatory sub-system, which includes the jaw, lip and tongue, provide sensitive markers of bulbar dysfunction (14). Measures of the extent and speed of lip and jaw movements predict a decline in speech intelligibility after 3 months in patients with ALS, while movement duration increased with disease progression (15, 16). In addition, measures of tongue movement duration and tongue movement speed differentiated between patients with advanced bulbar symptoms and healthy controls, while speed and size decreased with disease progression in early-stage bulbar patients (17). While these studies highlight the sensitivity of tongue kinematic features for the detection and tracking of bulbar impairment, the application of methods requires new and expensive equipment that is unavailable in most standard clinical settings. Therefore, these methods have not been adopted in standard clinical practice.

Building on this knowledge, this study aimed to develop a fast, automated and objective tool for quantifying bulbar kinematic features in patients by using equipment that is usually available in a clinical setting. We developed TT, a software that extracts tongue kinematic features from short, 5-s video clips of lateral tongue movements where the mouth is half-open. To use TT, it is only required to record tongue movements with a built-in camera by using a laptop, computer or mobile phone. TT is an open-source tool (link provided in the Methods section), which is platform-independent and can be optimized for different patient populations (e.g., stroke) or outcome variables (e.g., decreased movement performance over time). Here, we present and test our tool on ten patients with ALS and 16 age- and education-matched healthy controls. Two of the patients were clinically diagnosed with bulbar-onset type and eight patients were clinically diagnosed with (upper or lower) limb-onset type. In addition, we defined five limb-onset patients as bulbar-impaired by using cut-off scores on standard clinical assessments of bulbar function. We investigated whether the TT could differentiate between (i) bulbar-onset patients and healthy controls, (ii) bulbar-onset patients and limb-onset patients, (iii) bulbar-impaired limb-onset patients and healthy controls, and (iv) patients with ALS, regardless of onset type (bulbar and limb) and healthy controls. If differentiation was successful, our results would support the clinical relevance of tongue kinematic features for detecting and quantifying bulbar symptoms in patients with ALS with and without bulbar-onset type and would indicate that TT can serve as a useful tool to pursue that goal. Such measures could support the detection of bulbar symptoms as a marker of disease onset and progression for use in clinical trials.

## Materials and Methods

### Participants

Tongue movement videos were collected from patients with ALS (*n* = 17) and healthy control participants (*n* = 23). Several videos had to be excluded after quality inspection (see below for more details), leaving data of patients with ALS (*n* = 10) and healthy control participants (*n* = 16) for analysis. The demographic information for all the participants is shown in Table 1 and additional demographic information for patients is shown in Table 2. Patients with ALS were recruited from the Clinic for Neurology of the University Hospital of the Otto-von-Guericke University Magdeburg (SS), Hannover Medical School (SP) and Rostock University Medical Center (JP) in Germany. Criteria for selection of participants as healthy controls included no history of any neurological or psychiatric disease and the absence of sensorimotor impairments. The control group was age- and education-matched to the patient with ALS group at the group level (i.e., no significant difference between the groups in age and years of education, *p*-value below 0.05). Since all the patients and healthy controls also participated in a 7T-MRI study, they additionally had to meet 7T inclusion criteria, such as intact hearing (no tinnitus) and no tattoos or metal implants. All the participants provided informed consent and were paid seven euros per hour. This study was approved by the Ethics Committee of the University Hospital of the Otto-von-Guericke Universität Magdeburg (Ethics number: 16/17).

**Table 1:** Demographic variables for patients with amyotrophic lateral sclerosis (ALS) and healthy controls.

	**Patients (*n* = 10)**	**Controls (*n* = 16)**	**Group difference**		
	**Mean (SD)**	**Mean (SD)**	***df***	***t***	***Sig*** **.**
Age (years)	53 (16)	53 (23)	24	0.10	0.920
Education (years)	15 (3)	15 (3)	24	−0.80	0.431
Gender (M: F)	6:4	5:11	24	−0.145	0.161

*Group differences were measured by using the independent-samples t-tests*.

**Table 2:** Demographic information for patients with ALS.

**Patient ID**	**Onset type**	**Onset side**	**Phenotype**	**Age (years)**	**Sex**	**Education (years)**	**Height (cm)**	**Weight (kg)**	**El-Escorial**	**Disease duration (months)**
1	Lower limb	Left	UMND	74	F	15	176	92	Definite	13
2	Lower limb	Left	Classical ALS	36	M	16	168	64	Probable	26
3	Upper limb	Right	Classical ALS	48	M	13	185	100	Definite	186
4	Upper limb	Left	Classical ALS	61	M	15	180	85	Probable	11
5	Upper limb	Left	Classical ALS	66	M	18	180	74	Probable	11
6	Upper limb	Left	UMND	60	M	12	166	90	Definite	11
7	Upper limb	Left	LMND	52	F	12	158	60	Possible	18
8	Upper limb	Right	Classical ALS	20	F	12	160	90	Definite	2
9	Bulbar	Bulbar	LMND	64	F	13	162	59	Possible	6
10	Bulbar	Bulbar	UMND	53	M	21	179	77	Probable	33
Group mean (SD)				53 (16)		15 (3)	171 (10)	79 (15)		32 (55)

*UMND, Upper Motor Neuron Dominant; LMND, Lower Motor Neuron Dominant; El-Escorial, revised El-Escorial criteria*.

### Clinical Assessment

Table 3 provides an overview of the clinical information for the patients included in this study. Patients were classified according to the revised El-Escorial criteria (18) into one of the following categories: definite ALS, probable ALS or possible ALS by an experienced neurologist (SV) of the University Hospital Magdeburg, who also performed all the clinical assessments detailed below. Patients were also classified regarding onset type into either bulbar-onset type (*n* = 2) or (upper or lower) limb-onset type (*n* = 8) categories. Patients underwent a series of standard clinical tests including the ALSFRS-R (9), the CNS-BFS (11) and the Penn Upper Motor Neuron (UMN-Penn) score (19). Neither patients nor controls were tested for cognitive impairment.

**Table 3:** Clinical scores of patients with ALS.

**Patient ID**	**ALSFRS-R**	**ALSFRS-R bulbar**	**Disease progression rate**	**CNS-BFS**	**UMN-Penn**
1	25	7	1.8	53	26
2	45	12	0.1	22	15
3	37	12	0.1	21	2
4	41	11	0.6	29	6
5	41	9	0.6	31	2
6	40	10	0.7	32	11
7	42	12	0.3	22	16
8	14	8	17	28	-
9	47	11	0.2	28	1
10	33	7	0.5	62	2
Group mean (SD)	37 (10)	10 (2)	2.2 (5.2)	33 (14)	9 (9)

*Disease progression rate = (48-ALSFRS-R)/disease duration (months); ALSFRS-R, ALS functional rating scale-revised; UMN-Penn, Penn Upper Motor Neuron score; CNS-BFS, Center for Neurologic Study-Bulbar Function Scale; (– indicates missing data)*.

We defined patients as bulbar-impaired if they had the ALSFRS-R score equal to or below 11 or the CNS-BFS score above 43, as recommended for clinical use (11). Patients (*n* = 5) with limb-onset ALS were, thus, defined as bulbar-impaired and grouped separately from the two clinically diagnosed bulbar-onset patients. Disease duration was defined as the number of months between the onset of ALS symptoms and the time point of tongue measurement. Disease progression rate (DPR) was calculated according to the formula: DPR = (48-ALSFRS-R)/Disease duration (20).

### Experimental Task

**Figure 1:**
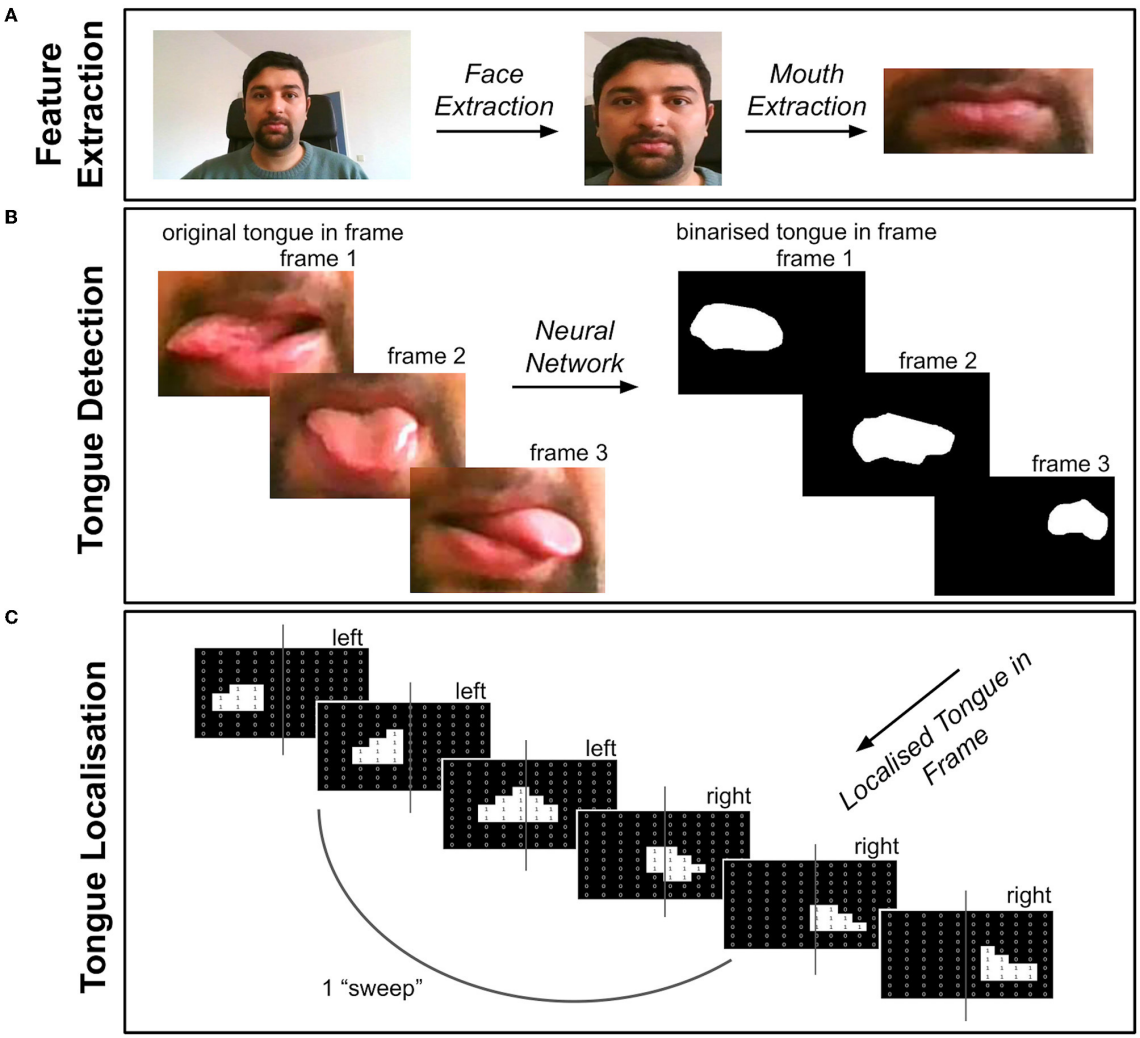
Overview of Tongue Tracker (TT). The videos were first pre-processed *via* steps detailed in the text (see pre-processing section). **(A)** The face and mouth were detected and extracted from each frame of the pre-processed videos. **(B)** The tongue was then detected by using a trained neural network, resulting in a binary image showing the location of the tongue in each video frame. **(C)** A record of the location of the tongue, either in the left or right portion of the frame, was recorded and saved for extracting kinematic features of the tongue. The code can be accessed here: https://github.com/BudhaTronix/Automated-Video-Analysis-Tool-for-Quantifying-Bulbar-Function.git.

Participants were seated in front of a laptop with a built-in camera. Three different models of Apple MacBook laptops with 720p cameras were used (MacBook Pro, 13-inch, 2017; MacBook Air, 13-inch, Mid 2012; MacBook Air, 13-inch, 2020), with which one of three total experimenters performed the test. Participants were given specific verbal instructions for the tongue movement task. Each participant was asked to open his/her mouth and move his/her tongue repeatedly between the two mouth commissures as fast as possible, while keeping the mouth half-open and while keeping the tongue visible, as given in Figure 1B. The movements were video recorded for a minimum of 5 s of correct movement. The task was completed twice to include one training run and one test run, resulting in two videos per participant. The second video was taken for further analysis, unless the video quality was rated as poor during pre-processing, in which case the first video was taken (this occurred for *n* = 5 participants). Such a simple experimental setting (i.e., without a second software to indicate onset and offset times or to give instructions) was chosen to make this task suitable for clinical routine. Healthy controls were tested at the German Center for Neurodegenerative Diseases (DZNE) in Magdeburg, whereas patients were tested at the University Clinic for Neurology within the University Hospital of the Otto-von-Guericke Universität Magdeburg.

### Video Quality Inspection

The inclusion criterion for the video clips was that the full face (including the mouth and the tongue) was visible in all the frames. This was important since TT first performs face detection and segmentation before it classifies tongue movements (see Figure 1). This criterion was ensured by two raters. Videos in which parts of the face or the eyes were not visible due to changes in camera angle or distance during the clip were, therefore, excluded from further analysis. Based on this criterion, healthy controls (*n* = 8) and patients with ALS (*n* = 7) had to be excluded from analysis. After exclusions, data from patients with ALS (*n* = 10) and healthy controls (*n* = 16) remained. To ensure that the full face is covered in the video clip, it is, therefore, recommended that the experimenter/clinician checks the video immediately after recording.

### Tongue Tracker

We developed a tongue tracking algorithm called TT, which uses the software Python to extract and quantify tongue movement features from video clips. Figure 1 provides an overview of each stage after pre-processing, including feature extraction, tongue movement detection and localization. The code is organized into separate modules (modules 1–5) for each processing stage and can be openly accessed here: https://github.com/BudhaTronix/Automated-Video-Analysis-Tool-for-Quantifying-Bulbar-Function.git. TT was developed on an HP Laptop with a Windows 10 operating system by using Pycharm with Python version 3.7 (21). It is an open-source tool and can be run on any operating system where Python 3.7 is installed. It is accessible through the command line but also *via* an easy-to-use graphical user interface (GUI). The user has an option to select whether the code should be run in single-mode (i.e., for one video) or in multi-mode (i.e., for all the videos in the local folder).

#### Pre-processing

The videos underwent pre-processing including trimming, stabilization and compression by using module 1 and module 2 of TT. First, the videos were trimmed to include the first 5 s only, to standardize the length of the videos across participants. The video stabilization module then minimized the effects of camera movement by tracking and estimating the motion of the features between two frames with the help of the Lucas–Kanade optical flow method in OpenCV (22). The videos were then compressed and resized to a standard aspect ratio by using the Moviepy package in the Python environment. Finally, the video files were converted from their original MOV format to standard MPEG format.

#### Face and Mouth Detection

The pre-processed videos then underwent feature extraction by using module 3 and module 4 of TT (see Figure 1A). The face was detected on a frame-by-frame basis by using Facenet, a pre-trained neural network in PyTorch (23). To extract the face in all the frames of the video, a custom function was created to add a bounding box around the detected face in each frame of the video. The coordinates of the bounding box in each frame were stored and the most extreme coordinates were used as a bounding box across all the frames, to ensure that the face was accurately extracted from every frame. The face-extracted videos were then passed through Facenet again, to detect and extract the mouth with a bounding box in each frame, as previously outlined. The mouth-extracted videos were then taken for further processing.

#### Neural Network

Since, to the best of our knowledge, there are no pre-trained models for tongue detection available online, we created a dataset to train a neural network for segmentation and detection of the tongue in each frame of the video (see Figure 1B). The dataset was created from the mouth-extracted videos. The videos were converted from a series of frames to images and the tongue masks were manually drawn by using ImageJ software for the purpose of training the neural network (24). The dataset, including the image and ground truth (i.e., binary image masks of the tongue segmented region), was then used to train the model. MobileNetV2 architecture (25) was used for tongue detection, since it is optimized for video analysis and supports faster detection and segmentation of the target. The neural network was pre-trained on ImageNet and then fine-tuned on the tongue dataset. After the network was trained, the tongue was detected automatically. Therefore, no manual intervention is required for using the final version of TT.

#### Extracting Tongue Movement Features

Using module 5 of TT, each frame of the video was passed through the new trained neural network to create a binary image showing the location of the tongue in each frame, as shown in Figure 1B. Using the midline of the frame, the location of the tongue in either the left or right side of the frame was recorded (see Figure 1C). This resulted in a location record of the tongue showing “L” for left or “R” for right, for each frame of the video.

Using the recorded locations of the tongue in each frame, we extracted the following tongue movement features. First, we calculated the total number of sweeps performed within the 5-s video clip. The lateral movement of the tongue back and forth between the corners of the mouth resulted in a typical location profile of multiple “R”s followed by multiple “L”s. A single sweep was defined as a tongue movement starting from halfway through a sequence of “R”s to halfway through a sequence of “L”s, depending on the location of the tongue at the start of the video. We took half of the sequence since the tongue moved from the center of the mouth to the corner before the next sweep began. We next calculated the average duration of a single sweep for each video by first calculating the number of frames taken for each sweep and then converting frames to seconds based on the known frame rate. Finally, we identified and recorded the number of errors in the tongue movement profiles. To achieve this, we first calculated the average number of frames in which the tongue was on the left and right sides of the frame. From this, we identified outliers where the tongue spent a longer than average time on one side of the frame, which could indicate an error made by the participant (i.e., lateral movement paused due to confusion regarding the direction of movement) or spasm due to involuntary muscle contractions.

### Validation

To validate the outputs of TT, an independent expert rater (MW) manually counted the number of sweeps performed within the analyzed video sequence without knowledge of the results of TT (i.e., first, the number of sweeps were counted manually before the automated numbers *via* TT were revealed).

### Statistical Analysis

To test the accuracy of TT in each group, we calculated correlations between the total number of sweeps identified by using TT and those identified by the independent expert rater for each group separately, using Pearson's correlation coefficient with a significance level of 5%. To test our hypotheses (see Introduction), we tested for group differences in tongue movement features (number of sweeps, average sweep duration, and number of errors) between each of the five bulbar-impaired limb-onset patients with healthy controls (one sample) using two-tailed, one-sample *t*-tests with a significance level of 5%. Additionally, we compared each of the two bulbar-onset patients with healthy controls (one sample) and limb-onset patients (one sample) by using two-tailed, one-sample *t*-tests with a significance level of 5%. Finally, we compared all the patients with healthy controls by using two-tailed, two-samples *t*-tests with a significance level of 5%. We report which analyses survived correction for multiple comparisons by using the Bonferroni correction.

## Results

### Validation Results

**Figure 2:**
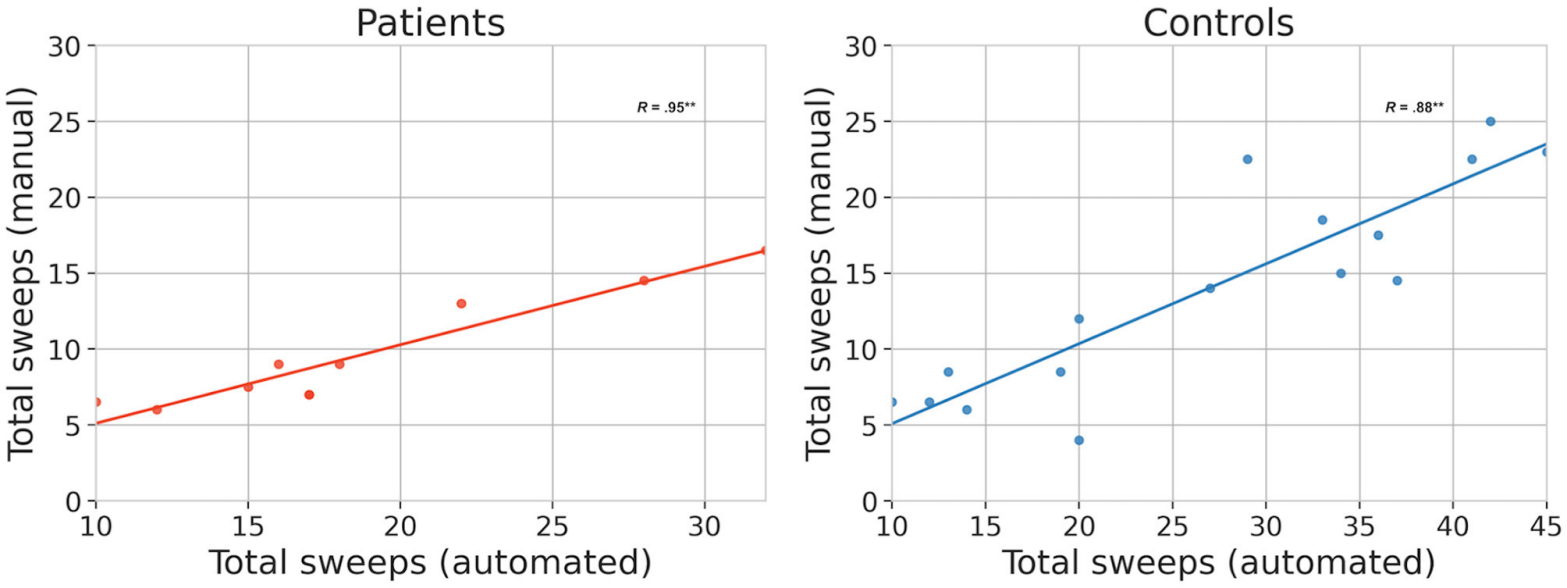
Validation results. Relationship between manual and automated (TT) methods for quantifying bulbar function based on the same video clips. The total number of sweeps are plotted separately for patients (*N* = 10) and healthy controls (*N* = 16). Correlations marked ^**^ are significant at the 5% level.

We correlated the total number of sweeps detected through manual counting with those from TT. The two measures were significantly correlated in both the patient group (*R* = 0.95^**^, *p* = 2.1 × 10^−5^) and the control group (*R* = 0.88^**^, *p* = 1 × 10^−5^), as shown in Figure 2.

### Tongue Kinematic Feature Results

**Figure 3:**
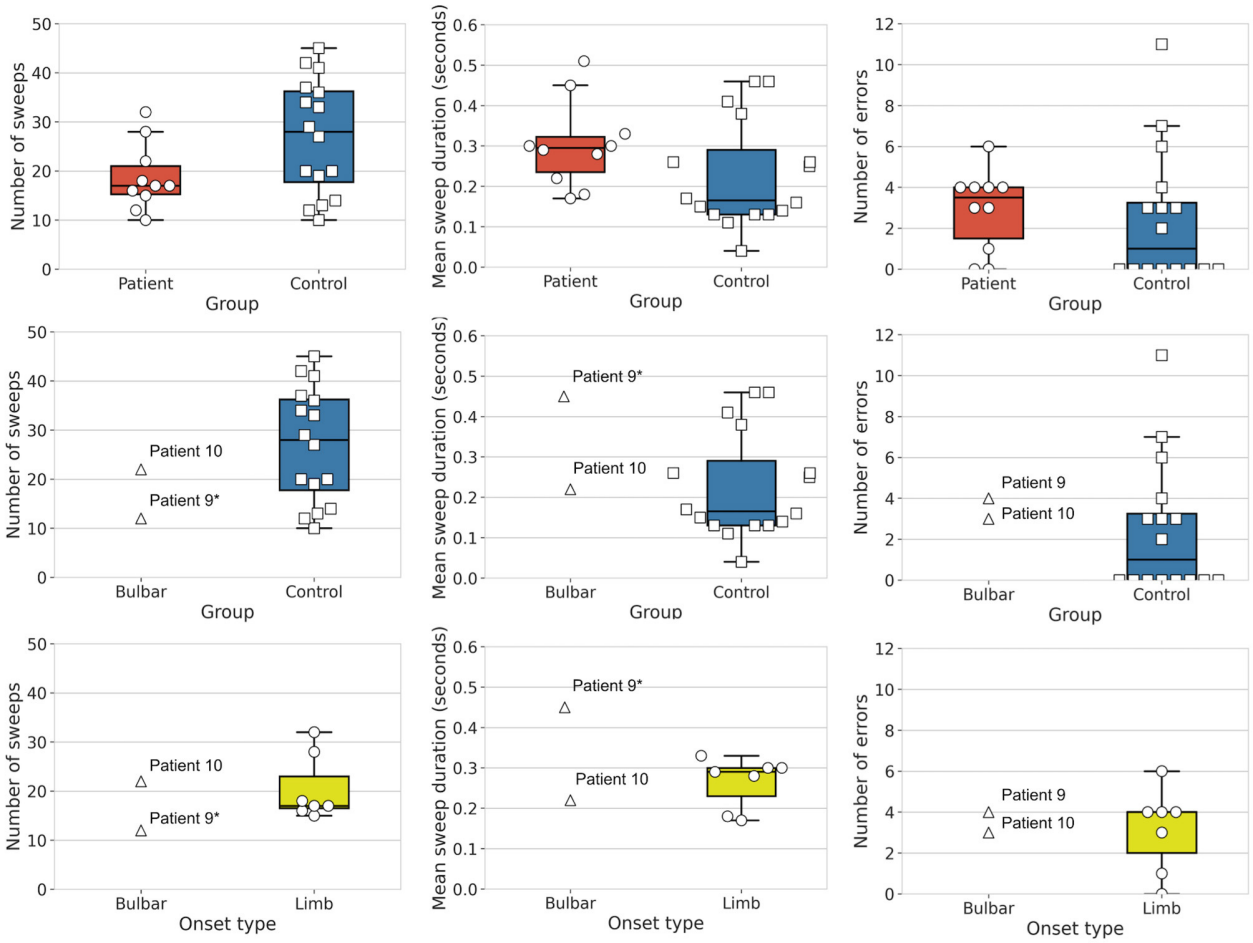
Group differences in tongue movement features. Shown are mean number of sweeps, mean sweep duration and mean number of errors. *Top row*: Box plots show patients with amyotrophic lateral sclerosis (ALS) (*N* = 10, circles, red) and healthy controls (*N* = 16, squares, blue). *Middle row*: Box plots show bulbar-onset patients (*N* = 2, triangles) and healthy controls (*N* = 16, squares, blue). *Bottom row*: Box plots show bulbar-onset patients (*N* = 2, triangles) and limb-onset patients (*N* = 16, circles, yellow), where limb-onset patients with bulbar impairment are shaded circles (Individual patients marked ^*^ are significant when compared to the healthy controls or limb-onset patients).

We tested for group differences in tongue kinematic features, for which detailed statistics are shown in Table 4. With respect to our first hypothesis [(i) differentiation between clinically diagnosed bulbar-onset patients with ALS (i.e., patient nine and patient ten) and healthy controls], as expected, patient nine performed significantly fewer sweeps with a significantly longer sweep duration compared to healthy controls, whereas, unexpectedly, patient ten did not show any significant differences in tongue kinematic features when compared to healthy controls. Patient nine also showed a trend toward more sweep errors compared to healthy controls (see Table 4 for statistics), as shown in Figure 3. With respect to our second hypothesis [(ii) differentiation between bulbar-onset patients with ALS (i.e., patient nine and patient ten) and limb-onset patients with ALS], patient nine again performed significantly fewer sweeps with a significantly longer duration compared to limb-onset patients, while patient ten showed a trend toward shorter sweep duration compared to limb-onset patients (see Table 4 for statistics), as shown in Figure 3.

**Table 4:** Overview over tongue kinematic features.

		**Group difference**			
		**Single patient value/mean across patients (SD) vs.- mean (SD) of comparison group**	***df***	***T***	***p*** **.**
Patient 1 vs. healthy controls (*N* = 16)	Number of sweeps	16 vs. 27 (11.71)	15	3.76	0.002^*^
	Average sweep duration	0.30 vs. 0.23 (.13)	15	−2.18	0.046^*^
	Number of errors	1 vs. 2.44 (3.25)	15	1.77	0.097^t^
Patient 4 vs. healthy controls (*N* = 16)	Number of sweeps	32 vs. 27 (11.71)	15	−1.71	0.108
	Average sweep duration	0.17 vs. 0.23 (.13)	15	1.73	0.105
	Number of errors	4 vs. 2.44 (3.25)	15	−1.93	0.073^t^
Patient 5 vs. healthy controls (*N* = 16)	Number of sweeps	17 vs. 27 (11.71)	15	3.42	0.004^*^
	Average sweep duration	0.30 vs. 0.23 (.13)	15	−2.18	0.046^*^
	Number of errors	3 vs. 2.44 (3.25)	15	−0.69	0.499
Patient 6 vs. healthy controls (*N* = 16)	Number of sweeps	18 vs. 27 (11.71)	15	3.02	0.008^*^
	Average sweep duration	0.28 vs. 0.23 (.13)	15	−1.58	0.136
	Number of errors	6 vs. 2.44 (3.25)	15	−4.39	*0.001* ^**^
Patient 8 vs. healthy controls (*N* = 16)	Number of sweeps	15 vs. 27 (11.71)	15	4.10	*0.001* ^**^
	Average sweep duration	0.33 vs. 0.23 (.13)	15	−3.08	0.008^*^
	Number of errors	4 vs. 2.44 (3.25)	15	−1.93	0.073^t^
Patient 9 vs. healthy controls (*N* = 16)	Number of sweeps	12 vs. 27 (11.71)	15	5.12	1.24 × 10^−4^^**^
	Average sweep duration	0.45 vs. 0.23 (.13)	15	−6.68	1 × 10^−5^^**^
	Number of errors	4 vs. 2.44 (3.25)	15	−1.93	0.073^t^
Patient 10 vs. healthy controls (*N* = 16)	Number of sweeps	22 vs. 27 (11.71)	15	1.71	0.110
	Average sweep duration	0.22 vs. 0.23 (.13)	15	0.22	0.825
	Number of errors	3 vs. 2.44 (3.25)	15	−0.69	0.499
Patient 9 vs. limb-onset patients (*N* = 8)	Number of sweeps	12 vs. 19.13 (7.22)	7	2.79	0.027^*^
	Average sweep duration	0.45 vs. 0.30 (.13)	7	−4.19	0.004^*^
	Number of errors	4 vs. 2.75 (2.19)	7	−1.62	0.150
Patient 10 vs. limb-onset patients (*N* = 8)	Number of sweeps	22 vs. 19.13 (7.22)	7	−1.13	0.297
	Average sweep duration	0.22 vs. 0.30 (.13)	7	2.03	0.082
	Number of errors	3 vs. 2.75 (2.19)	7	−0.32	0.756
All patients (*N* = 10) vs. healthy controls (*N* = 16)^∧^	Number of sweeps	18.70 (6.85) vs. 27 (11.71)	24	2.03	0.050^t^
	Average sweep duration	0.30 (.11) vs. 0.23 (.13)	24	−1.51	0.150
	Number of errors	2.90 (1.97) vs. 2.44 (3.25)	24	−0.41	0.690

*Four types of comparisons were made: (i) individual bulbar-impaired limb-onset patients with ALS (patients 1, 4, 5, 6, and 8) and healthy controls (lines 1–5), (ii) individual bulbar-onset patients with ALS (patients 9 and 10) and healthy controls (lines 6 and 7), (iii) individual bulbar-onset patients with ALS (patients 9 and 10) and limb-onset patients with ALS (lines 8 and 9), and (iv) all the patients with ALS (regardless of onset type) and healthy controls (line 10). Statistics are reported as one-sample t-tests and two-sample t-tests*

*(^∧^ indicates use of two-sample t-tests); those marked*

* *and*

** *are significant at the 5 and 1% uncorrected level, respectively; those marked in bold remained significant at the Bonferroni-corrected threshold of p < 0.0024); differences marked ^t^ are trending significance (i.e., Sig. < 0.1)*.

With respect to our third hypothesis [(iii) differentiation between bulbar-impaired limb-onset patients with ALS (i.e., patients one, four, five, six and eight) and healthy controls], as expected, patients one, five, six and eight performed significantly fewer sweeps compared to healthy controls (see Table 4 for statistics), while patient four did not show a significant difference compared to healthy controls. Patients one, five and eight performed significantly slower tongue sweeps compared to healthy controls, while patients four and six did not show a significant difference when compared to healthy controls. With respect to the number of errors, patient six showed significantly more errors compared to healthy controls, while patient five did not show a significant difference in number of errors compared to healthy controls. Patients four and eight showed a trend toward more errors compared to healthy controls, while patient one showed a trend toward fewer errors compared to healthy controls (see Table 4 for statistics).

With respect to our fourth hypothesis [(iv) differentiation between patients with ALS regardless of onset type and healthy controls], the group of patients with ALS showed a statistical trend toward completing fewer sweeps compared to the group of controls, while there were no significant differences and no trend toward a significant difference in sweep duration or in sweep errors in the patient group compared to healthy controls (see Table 4 for statistics), as shown in Figure 3.

## Discussion

We present a novel and open-access tool called TT that automatically quantifies tongue kinematic features from video clips of lateral tongue movements. We tracked tongue kinematic features, namely, the number of tongue sweeps, tongue movement duration, and tongue movement errors, in healthy controls and in a heterogeneous group of patients with ALS, some of whom presented with bulbar symptoms and some of whom showed mainly limb-associated symptoms. One of the two patients with bulbar-onset ALS had a significantly different tongue kinematic profile compared to both the group of healthy controls and compared to the limb-onset patients with respect to the number of tongue sweeps and average sweep duration. The second bulbar-onset patient, who suffered from ALS for many years without rapid disease progression, did not show such a distinct profile. In the group of limb-onset patients with bulbar impairment, four patients showed significantly different tongue kinematic profiles when compared to healthy controls with respect to a number of tongue sweeps. Overall, the group of patients with ALS showed a statistical trend toward fewer and slower tongue movements compared to healthy controls. Our open-source tool provides a new, quantitative measure of bulbar dysfunction for use in ALS and possibly other neurological disorders presenting with dysphagia that can be used to identify dysfunction and to track bulbar function over time.

We first investigated how TT characterizes the tongue movements of patients with a clinical diagnosis of bulbar-onset type ALS. We, therefore, compared each of the two cases of bulbar-onset patients with ALS with both the healthy controls and the limb-onset patients. Patient nine showed a tongue kinematic profile in line with reduced bulbar function (i.e., significantly fewer and slower tongue movements), whereas patient ten did not show such a profile. There is a clear difference in the disease etiology of these two patients. Patient nine was in the early disease stage at only 3 months post-diagnosis, whereas patient ten had been diagnosed 33 months prior to this study. Despite this long disease duration, patient 10 was able to complete 1 h of MRI scanning, suggesting a slow disease progression. This difference in disease stage is reflected by the patients' clinical scores on the CNS-BFS, the ALSFRS-R and the ALSFRS-R bulbar sub-score, in which patient 10 showed greater impairment on all of the scales. Research has highlighted a potential compensatory mechanism in which patients in the advanced stages of ALS show increased jaw movements to compensate for decreased tongue movement speed and range in speech, before they then present with more severe symptoms when compensation fails (17, 26). This compensatory mechanism in advanced bulbar ALS, which may particularly occur when the disease progresses slowly, may account for the trend toward faster tongue movements when patient ten was compared to limb-onset patients. In this respect, it would be interesting in future studies to also record and analyse jaw movements in the videos.

While bulbar function is supported bilaterally in the healthy brain, disease onset in ALS is typically lateralised to one hemisphere and seems to spread differently through the lower motor neurons in comparison to the primary motor cortex (6). We speculate that in bulbar-onset patients with greater UMN involvement, tongue kinematic features may be most impacted in the early disease stage. With disease progression, a slower decline or even improvement in performance may occur due to compensation from both the non-affected hemisphere and non-affected jaw muscles. At the more advanced disease stages, we would expect tongue kinematic features to decline again as compensation is inhibited by more severe and bilateral bulbar impairment. The two bulbar-onset patients in this study differed in their motor neuron involvement, where patient 9 has a lower motor neuron dominant phenotype and patient 10 has an UMN dominant phenotype. This difference in phenotype may explain why the tongue kinematic profile of patient 10 could not be distinguished from healthy controls, where patient 10 may have been in the stage of compensation as mentioned above.

Next, we investigated the sensitivity of TT to detect bulbar symptoms in limb-onset patients. We, therefore, compared each of the five bulbar-impaired limb-onset patients with ALS to the healthy controls. Four out of five bulbar-impaired limb-onset patients showed fewer tongue movements compared to controls and three out of five bulbar-impaired limb-onset patients also showed significantly slower tongue movements compared to controls. Out of the two patients who did not show significant differences in movement duration compared to controls, patient six showed significantly more errors, while patient four showed a trend toward more errors in tongue movement compared to healthy controls. This may reflect a speed-accuracy trade-off in both the patients. Since a similar tongue kinematic profile was reflected in the bulbar-onset patients (see above), we think that, in particular, the number of sweeps and sweep duration, rather than the number of errors, are sensitive markers to quantify bulbar impairment in patients with ALS. Such features likely reflect the signature spasticity of the tongue associated with bulbar impairment caused by UMN degeneration, which has been shown to independently predict survival time in patients with ALS (27). This is also supported by previous studies in which speech-based measures identified early bulbar symptoms undetected by both the patient self-reports and clinician speech ratings (12) and where lip and jaw kinematic features predicted a decline in speech intelligibility by 3 months (15).

Next, we also compared the full, heterogeneous group of patients with ALS to healthy controls to see if TT could differentiate between patients and controls. Here, TT detected a trend toward patients with ALS completing fewer tongue sweeps compared to healthy controls. Our sample of patients with ALS was highly heterogeneous in regards to disease duration and disease progression rate and also with respect to the involvement of bulbar impairments. Even though this trend may be significant when testing the larger patient groups, TT may be more suitable for identifying bulbar impairments in the patient groups rather than differentiating between patients with ALS and controls.

We think that TT may also be particularly suitable to track changes in tongue kinematic features in limb-onset patients as they appear when the disease spreads to the bulbar area of the primary motor cortex (M1) over time (6). To test for these assumptions, one could perform longitudinal sampling with our tool to track tongue kinematic features with disease progression. Accurately quantifying bulbar dysfunction is further necessary to identify corresponding brain changes to the bulbar area of M1 by using neuroimaging methods (7, 28). In addition, our measure could be used to track tongue movement training success to improve outcomes in dysphagia, as previously demonstrated with specialized equipment to measure tongue pressure in patients following acquired brain injury and stroke (4). While we did not directly compare the patients with bulbar symptoms to a disease control group (e.g., stroke patients with bulbar symptoms), we expect a differentiation because the performance of patients with ALS should worsen over time, even after a possible compensation period as the disease progresses further due to continuous neurodegeneration.

The current diagnosis of bulbar dysfunction in ALS is clinical and, thus, requires assessment by an expert neurologist to identify subtle signs, particularly in the early stages of the disease. A key advantage of TT is that it requires neither new equipment nor expert medical knowledge to extract information regarding the kinematic features of the tongue. Instead of using expensive sensors to estimate camera motion, we pre-processed the videos to reduce motion and background noise. We think that TT is also relevant for clinicians with expertise in ALS, since it can quantify bulbar impairment reliably over time with different examiners. Along with our open-access code, we also provide all the tongue video clips to enable other researchers to fine-tune the model as per their needs. For example, one could extend the code to track the jaw movement, which could be used to test the compensation hypothesis in ALS (see above).

One limitation of this study is the relatively small sample size of patients with ALS. Given the low prevalence of ALS in the general population, availability of patients is generally poor. With our relatively low sample size, the investigation of sub-groups was limited and some statistical evaluations, such as disease classification or prediction, could not be calculated based on our dataset. Future studies may apply our tool in large-cohort analyses to reveal more information about sub-group classification. In addition, this study did not directly compare the results of TT with invasive, quantitative measures of tongue movement or swallowing function. It would be important to compare the reliability of the different tools to quantify bulbar impairment. Future studies may, therefore, investigate a multitude of parallel measures to provide recommendations as to which tool is most suitable in different disease stages.

Given the signature spasticity symptoms of UMN degeneration, our measure likely largely reflects UMN involvement in bulbar impairment. We suggest that the addition of a still tongue task along with modifications to the motion tracking code could allow for measurement of tongue fasciculations, which may provide important insight into lower motor neuron involvement. Since we had a high exclusion rate of videos, we recommend that future users of TT position the camera directly in front of the participant and not below, to ensure that the full face, including eyes and forehead, is visible. This is necessary for successful face detection and tracking during pre-processing. While we did not test the impact of cognitive impairment on TT, we suggest that TT would be equally successful with additional training time and/or when using visual instructions *via* photos or short instruction videos. Since we did not have the disease control group, for example, a group of patients with non-ALS with bulbar symptoms (e.g., patients with stroke or patients with Parkinson's disease), we cannot make any conclusions about the utility of TT for the other patient groups.

In conclusion, we here introduce TT, a fast, quantitative and open-access tool for quantifying bulbar function and provide first clinical data in a small sample of patients with ALS and healthy control participants. We found that the tongue kinematic features, in particular the number and duration of tongue movements, can identify some patients with bulbar symptoms with or without a diagnosis of bulbar-onset type. Such features likely reflect the signature spasticity and weakness of the tongue associated with bulbar impairment, caused by UMNs. Our open-source tool may serve as a quantitative marker for symptoms of bulbar dysfunction in other disorders beyond ALS, such as stroke.

## Data Availability Statement

The raw data supporting the conclusions of this article will be made available by the authors, without undue reservation.

## Ethics Statement

The studies involving human participants were reviewed and approved by Ethics Committee of the University Clinic Magdeburg. Number: 16/17. The patients/participants provided their written informed consent to participate in this study. Written informed consent was obtained from the individual(s) for the publication of any potentially identifiable images or data included in this article.

## Author Contributions

This study was designed by EK, MW, and SS. Patients were recruited by SS, SP, and JP. Data collection was performed by MW and AN. Clinical assessment was performed by SV. Formal analysis was completed by BM and AN. Data investigation and statistical analysis were completed by AN. The first draft of the manuscript was written by AN. The manuscript was reviewed and edited by MW, EK, SS, and BM. This study was supervised by EK and SS. All authors contributed to the article and approved the submitted version.

## Funding

This project was funded by Else Kröner-Fresenius-Stiftung (EKFS) (Grant Number 2019-A03).

## Conflict of Interest

The authors declare that the research was conducted in the absence of any commercial or financial relationships that could be construed as a potential conflict of interest.

## Publisher's Note

All claims expressed in this article are solely those of the authors and do not necessarily represent those of their affiliated organizations, or those of the publisher, the editors and the reviewers. Any product that may be evaluated in this article, or claim that may be made by its manufacturer, is not guaranteed or endorsed by the publisher.
